# (*E*)-*N*′-(5-Bromo-2-methoxy­benzyl­idene)-4-methoxy­benzohydrazide

**DOI:** 10.1107/S1600536808035472

**Published:** 2008-11-08

**Authors:** Hong-Yan Ban, Cong-Ming Li

**Affiliations:** a School of Chemical Engineering, University of Science and Technology Liaoning, Anshan 114051, People’s Republic of China; b College of Sciences, Shenyang University, Shenyang 110044, People’s Republic of China

## Abstract

In the title compound, C_16_H_15_BrN_2_O_3_, the benzohydrazide group is not planar and the mol­ecule exists in a *trans* configuration with respect to the methyl­idene unit. The dihedral angle between the two substituted benzene rings is 75.6 (2)°. In the crystal structure, mol­ecules are linked by inter­molecular N—H⋯O hydrogen bonds involving carbonyl and amine functionalities, to form chains parallel to the *c* cell axis.

## Related literature

For the biological activities of hydrazones, see: Zhong *et al.* (2007 ▶); Raj *et al.* (2007 ▶); Jimenez-Pulido *et al.* (2008 ▶). For related structures, see: Yehye *et al.* (2008 ▶); Fun, Patil, Jebas *et al.* (2008 ▶); Fun, Patil, Rao *et al.* (2008 ▶); Yang *et al.* (2008 ▶); Ejsmont *et al.* (2008 ▶).
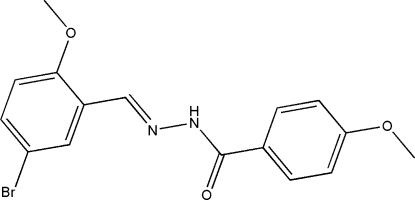


## Experimental

### 

#### Crystal data

C_16_H_15_BrN_2_O_3_
*M* *_r_* = 363.21
Monoclinic, 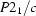

*a* = 12.438 (4) Å
*b* = 16.684 (6) Å
*c* = 7.863 (3) Å
β = 108.218 (6)°
*V* = 1549.8 (9) Å^3^
*Z* = 4
Mo *K*α radiation
μ = 2.67 mm^−1^
*T* = 298 (2) K
0.20 × 0.20 × 0.18 mm

#### Data collection

Bruker SMART CCD area-detector diffractometer
Absorption correction: multi-scan (*SADABS*; Bruker, 1998 ▶) *T*_min_ = 0.593, *T*_max_ = 0.620
8697 measured reflections
3245 independent reflections
2068 reflections with *I* > 2σ(*I*)
*R*_int_ = 0.034

#### Refinement

*R*[*F* ^2^ > 2σ(*F* ^2^)] = 0.038
*wR*(*F* ^2^) = 0.093
*S* = 1.01
3245 reflections
205 parameters
1 restraint
H atoms treated by a mixture of independent and constrained refinement
Δρ_max_ = 0.50 e Å^−3^
Δρ_min_ = −0.47 e Å^−3^

### 

Data collection: *SMART* (Bruker, 1998 ▶); cell refinement: *SAINT* (Bruker, 1998 ▶); data reduction: *SAINT*; program(s) used to solve structure: *SHELXS97* (Sheldrick, 2008 ▶); program(s) used to refine structure: *SHELXL97* (Sheldrick, 2008 ▶); molecular graphics: *SHELXTL* (Sheldrick, 2008 ▶); software used to prepare material for publication: *SHELXTL*.

## Supplementary Material

Crystal structure: contains datablocks global, I. DOI: 10.1107/S1600536808035472/bh2202sup1.cif

Structure factors: contains datablocks I. DOI: 10.1107/S1600536808035472/bh2202Isup2.hkl

Additional supplementary materials:  crystallographic information; 3D view; checkCIF report

## Figures and Tables

**Table 1:** Hydrogen-bond geometry (Å, °)

*D*—H⋯*A*	*D*—H	H⋯*A*	*D*⋯*A*	*D*—H⋯*A*
N2—H2⋯O2^i^	0.890 (10)	1.966 (12)	2.835 (3)	165 (2)

Symmetry code: (i) 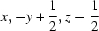.

**Fig. 1.**
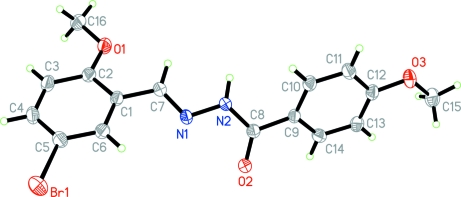
The molecular structure of (I), showing 30% probability displacement ellipsoids for the non-hydrogen atoms.

**Fig. 2.**
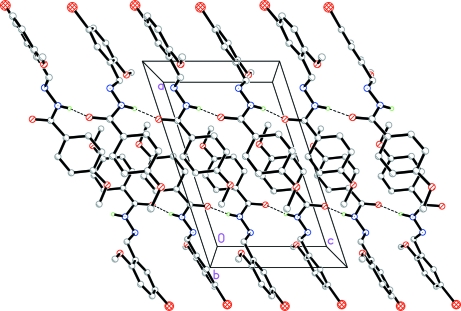
The packing diagram of (I), viewed along the b axis. Hydrogen bonds are shown as dashed lines.

## supplementary crystallographic information

### Comment

Hydrazones derived from the condensation of aldehydes with hydrazides have been demonstrated to possess excellent biological activities (Zhong *et al.*, 2007; Raj *et al.*, 2007; Jimenez-Pulido *et al.*, 2008). Due to the easy synthesis of such compounds, a great deal of hydrazones have been synthesized and structurally characterized (Yehye *et al.*, 2008; Fun, Patil, Jebas *et al.*, 2008; Fun, Patil, Rao *et al.*, 2008;Yang *et al.*, 2008; Ejsmont *et al.*, 2008). We report herein the crystal structure of the title new compound, (I).

In the structure of the title compound (Fig. 1) the molecule exists in a *trans* configuration with respect to the methylidene unit. The dihedral angle between the two substituted benzene rings is 75.6 (2)°. In both the 5-bromo-2-methoxyphenyl unit and the 4-methoxyphenyl unit, the methoxy groups are nearly coplanar with the corresponding mean planes of the C1···C6 and C9···C14 rings, respectively. Atoms C16 and C15 deviate from their corresponding benzene rings by 0.058 (2) and 0.059 (2) Å respectively. The torsion angle of C7—N1—N2—C8 is 19.4 (3)°. The bond lengths and angles are found in expected ranges.

In the crystal structure, molecules are linked by intermolecular N—H···O hydrogen bonds involving carbonyl and amine groups (Table 1), to form chains parallel to the *c* axis (Fig. 2).

### Experimental

The compound was prepared by refluxing 5-bromo-2-methoxybenzaldehyde (1.0 mmol) with 4-methoxybenzohydrazide (1.0 mmol) in methanol (100 ml). Excess methanol was removed from the mixture by distillation. The colorless solid product was filtered, and washed three times with methanol. Colorless block crystals of the title compound were obtained from a methanol solution by slow evaporation in air.

### Refinement

H2 was located in a difference map and refined isotropically, with N2—H2 distance restrained to 0.90 (1) Å. Other H atoms were placed in calculated positions (C—H = 0.93–0.96 Å) and refined as riding atoms with *U*_iso_(H) = 1.2*U*_eq_(C) and 1.5*U*_eq_(methyl C). A rotating group model was used for the methyl groups.

### Crystal data

**Table:**

C_16_H_15_BrN_2_O_3_	*F*_000_ = 736
*M**_r_* = 363.21	*D*_x_ = 1.557 Mg m^−^^3^
Monoclinic, *P*2_1_/*c*	Mo *K*α radiation λ = 0.71073 Å
Hall symbol: -P 2ybc	Cell parameters from 2110 reflections
*a* = 12.438 (4) Å	θ = 2.4–24.5º
*b* = 16.684 (6) Å	µ = 2.67 mm^−^^1^
*c* = 7.863 (3) Å	*T* = 298 (2) K
β = 108.218 (6)º	Block, colorless
*V* = 1549.8 (9) Å^3^	0.20 × 0.20 × 0.18 mm
*Z* = 4	

### Data collection

**Table:**

Bruker SMART CCD area-detector diffractometer	3245 independent reflections
Radiation source: fine-focus sealed tube	2068 reflections with *I* > 2σ(*I*)
Monochromator: graphite	*R*_int_ = 0.034
*T* = 298(2) K	θ_max_ = 26.7º
ω scans	θ_min_ = 1.7º
Absorption correction: multi-scan(SADABS; Bruker, 1998)	*h* = −14→15
*T*_min_ = 0.593, *T*_max_ = 0.620	*k* = −20→21
8697 measured reflections	*l* = −9→9

### Refinement

**Table:**

Refinement on *F*^2^	Secondary atom site location: difference Fourier map
Least-squares matrix: full	Hydrogen site location: inferred from neighbouring sites
*R*[*F*^2^ > 2σ(*F*^2^)] = 0.038	H atoms treated by a mixture of independent and constrained refinement
*wR*(*F*^2^) = 0.093	*w* = 1/[σ^2^(*F*_o_^2^) + (0.0407*P*)^2^ + 0.1883*P*] where *P* = (*F*_o_^2^ + 2*F*_c_^2^)/3
*S* = 1.01	(Δ/σ)_max_ = 0.001
3245 reflections	Δρ_max_ = 0.50 e Å^−^^3^
205 parameters	Δρ_min_ = −0.47 e Å^−^^3^
1 restraint	Extinction correction: none
Primary atom site location: structure-invariant direct methods	

### Fractional atomic coordinates and isotropic or equivalent isotropic displacement parameters (Å^2^)

**Table:**

	*x*	*y*	*z*	*U*_iso_*/*U*_eq_	
Br1	−0.31436 (3)	0.22534 (2)	0.94364 (5)	0.07161 (17)	
N1	0.10628 (17)	0.23762 (13)	0.8539 (3)	0.0380 (5)	
N2	0.19688 (18)	0.26531 (13)	0.8053 (3)	0.0384 (5)	
O1	0.01300 (15)	0.01461 (11)	0.7292 (3)	0.0509 (5)	
O2	0.26258 (15)	0.33509 (11)	1.0641 (2)	0.0455 (5)	
O3	0.61120 (16)	0.44804 (12)	0.6741 (3)	0.0603 (6)	
C1	−0.0401 (2)	0.14085 (16)	0.8049 (3)	0.0383 (6)	
C2	−0.0634 (2)	0.05851 (16)	0.7802 (3)	0.0414 (7)	
C3	−0.1589 (2)	0.02734 (18)	0.8110 (4)	0.0490 (7)	
H3	−0.1738	−0.0273	0.7967	0.059*	
C4	−0.2318 (2)	0.07627 (19)	0.8625 (4)	0.0498 (8)	
H4	−0.2957	0.0548	0.8828	0.060*	
C5	−0.2101 (2)	0.15743 (18)	0.8841 (4)	0.0460 (7)	
C6	−0.1148 (2)	0.18901 (17)	0.8578 (3)	0.0429 (7)	
H6	−0.1000	0.2435	0.8757	0.051*	
C7	0.0603 (2)	0.17374 (16)	0.7739 (3)	0.0389 (6)	
H7	0.0915	0.1481	0.6953	0.047*	
C8	0.2703 (2)	0.31655 (15)	0.9165 (4)	0.0369 (6)	
C9	0.3602 (2)	0.34885 (15)	0.8498 (3)	0.0354 (6)	
C10	0.3494 (2)	0.35720 (16)	0.6705 (4)	0.0425 (7)	
H10	0.2833	0.3400	0.5852	0.051*	
C11	0.4338 (2)	0.39027 (18)	0.6149 (4)	0.0493 (7)	
H11	0.4246	0.3954	0.4934	0.059*	
C12	0.5326 (2)	0.41596 (16)	0.7408 (4)	0.0432 (7)	
C13	0.5452 (2)	0.40845 (18)	0.9203 (4)	0.0482 (7)	
H13	0.6115	0.4256	1.0054	0.058*	
C14	0.4593 (2)	0.37549 (17)	0.9738 (4)	0.0462 (7)	
H14	0.4681	0.3711	1.0953	0.055*	
C15	0.7161 (3)	0.4720 (2)	0.7978 (5)	0.0739 (11)	
H15A	0.7031	0.5129	0.8749	0.111*	
H15B	0.7644	0.4926	0.7338	0.111*	
H15C	0.7517	0.4267	0.8684	0.111*	
C16	−0.0095 (3)	−0.06880 (17)	0.6972 (4)	0.0563 (8)	
H16A	−0.0110	−0.0943	0.8059	0.084*	
H16B	0.0488	−0.0925	0.6575	0.084*	
H16C	−0.0814	−0.0757	0.6068	0.084*	
H2	0.212 (2)	0.2407 (14)	0.715 (3)	0.044 (8)*	

### Atomic displacement parameters (Å^2^)

**Table:**

	*U*^11^	*U*^22^	*U*^33^	*U*^12^	*U*^13^	*U*^23^
Br1	0.0574 (2)	0.0821 (3)	0.0863 (3)	0.00286 (18)	0.0383 (2)	−0.0118 (2)
N1	0.0331 (12)	0.0430 (14)	0.0405 (13)	−0.0059 (10)	0.0154 (10)	−0.0001 (10)
N2	0.0398 (13)	0.0409 (13)	0.0403 (14)	−0.0086 (11)	0.0208 (11)	−0.0065 (11)
O1	0.0498 (12)	0.0413 (11)	0.0670 (14)	−0.0080 (9)	0.0260 (11)	−0.0072 (10)
O2	0.0488 (11)	0.0473 (11)	0.0470 (12)	−0.0117 (9)	0.0244 (10)	−0.0083 (9)
O3	0.0472 (13)	0.0689 (14)	0.0728 (15)	−0.0201 (11)	0.0300 (11)	−0.0025 (12)
C1	0.0363 (15)	0.0414 (16)	0.0361 (15)	−0.0074 (12)	0.0098 (13)	−0.0008 (12)
C2	0.0401 (16)	0.0453 (17)	0.0371 (16)	−0.0058 (13)	0.0098 (13)	−0.0017 (13)
C3	0.0483 (17)	0.0462 (17)	0.0525 (19)	−0.0146 (14)	0.0158 (15)	−0.0021 (14)
C4	0.0411 (17)	0.060 (2)	0.0503 (18)	−0.0150 (15)	0.0176 (15)	0.0009 (15)
C5	0.0404 (16)	0.058 (2)	0.0410 (17)	−0.0019 (14)	0.0145 (14)	0.0009 (14)
C6	0.0437 (16)	0.0433 (16)	0.0412 (17)	−0.0049 (13)	0.0125 (14)	−0.0019 (13)
C7	0.0372 (15)	0.0417 (16)	0.0404 (16)	−0.0035 (13)	0.0158 (13)	−0.0020 (13)
C8	0.0368 (15)	0.0318 (14)	0.0436 (17)	0.0025 (12)	0.0147 (13)	0.0013 (12)
C9	0.0328 (14)	0.0328 (14)	0.0422 (17)	−0.0033 (11)	0.0142 (13)	−0.0010 (12)
C10	0.0350 (15)	0.0479 (17)	0.0422 (17)	−0.0075 (13)	0.0086 (13)	−0.0016 (13)
C11	0.0504 (18)	0.0560 (19)	0.0450 (17)	−0.0098 (14)	0.0201 (15)	0.0023 (14)
C12	0.0365 (16)	0.0385 (15)	0.057 (2)	−0.0043 (12)	0.0184 (15)	−0.0021 (13)
C13	0.0340 (16)	0.0556 (19)	0.054 (2)	−0.0106 (13)	0.0128 (14)	−0.0093 (15)
C14	0.0453 (17)	0.0554 (18)	0.0400 (17)	−0.0063 (14)	0.0165 (14)	−0.0055 (14)
C15	0.048 (2)	0.087 (3)	0.096 (3)	−0.0268 (18)	0.036 (2)	−0.017 (2)
C16	0.062 (2)	0.0428 (18)	0.067 (2)	−0.0070 (15)	0.0239 (17)	−0.0084 (15)

### Geometric parameters (Å, °)

**Table:**

Br1—C5	1.888 (3)	C6—H6	0.9300
N1—C7	1.278 (3)	C7—H7	0.9300
N1—N2	1.378 (3)	C8—C9	1.477 (3)
N2—C8	1.352 (3)	C9—C10	1.381 (4)
N2—H2	0.890 (10)	C9—C14	1.385 (4)
O1—C2	1.355 (3)	C10—C11	1.372 (4)
O1—C16	1.426 (3)	C10—H10	0.9300
O2—C8	1.233 (3)	C11—C12	1.383 (4)
O3—C12	1.355 (3)	C11—H11	0.9300
O3—C15	1.419 (4)	C12—C13	1.377 (4)
C1—C6	1.386 (4)	C13—C14	1.379 (4)
C1—C2	1.405 (4)	C13—H13	0.9300
C1—C7	1.453 (3)	C14—H14	0.9300
C2—C3	1.386 (4)	C15—H15A	0.9600
C3—C4	1.371 (4)	C15—H15B	0.9600
C3—H3	0.9300	C15—H15C	0.9600
C4—C5	1.381 (4)	C16—H16A	0.9600
C4—H4	0.9300	C16—H16B	0.9600
C5—C6	1.371 (4)	C16—H16C	0.9600
			
C7—N1—N2	115.0 (2)	C10—C9—C14	117.8 (2)
C8—N2—N1	118.5 (2)	C10—C9—C8	123.9 (2)
C8—N2—H2	122.6 (17)	C14—C9—C8	118.2 (2)
N1—N2—H2	117.5 (17)	C11—C10—C9	121.8 (3)
C2—O1—C16	117.6 (2)	C11—C10—H10	119.1
C12—O3—C15	117.8 (2)	C9—C10—H10	119.1
C6—C1—C2	118.7 (2)	C10—C11—C12	119.6 (3)
C6—C1—C7	121.5 (2)	C10—C11—H11	120.2
C2—C1—C7	119.8 (2)	C12—C11—H11	120.2
O1—C2—C3	124.6 (3)	O3—C12—C13	124.6 (2)
O1—C2—C1	115.9 (2)	O3—C12—C11	115.6 (3)
C3—C2—C1	119.5 (3)	C13—C12—C11	119.7 (3)
C4—C3—C2	120.7 (3)	C12—C13—C14	119.9 (3)
C4—C3—H3	119.7	C12—C13—H13	120.0
C2—C3—H3	119.7	C14—C13—H13	120.0
C3—C4—C5	119.9 (3)	C13—C14—C9	121.2 (3)
C3—C4—H4	120.0	C13—C14—H14	119.4
C5—C4—H4	120.0	C9—C14—H14	119.4
C6—C5—C4	120.2 (3)	O3—C15—H15A	109.5
C6—C5—Br1	120.0 (2)	O3—C15—H15B	109.5
C4—C5—Br1	119.8 (2)	H15A—C15—H15B	109.5
C5—C6—C1	121.0 (3)	O3—C15—H15C	109.5
C5—C6—H6	119.5	H15A—C15—H15C	109.5
C1—C6—H6	119.5	H15B—C15—H15C	109.5
N1—C7—C1	120.5 (2)	O1—C16—H16A	109.5
N1—C7—H7	119.7	O1—C16—H16B	109.5
C1—C7—H7	119.7	H16A—C16—H16B	109.5
O2—C8—N2	122.1 (2)	O1—C16—H16C	109.5
O2—C8—C9	122.2 (2)	H16A—C16—H16C	109.5
N2—C8—C9	115.7 (2)	H16B—C16—H16C	109.5

### Hydrogen-bond geometry (Å, °)

**Table:**

*D*—H···*A*	*D*—H	H···*A*	*D*···*A*	*D*—H···*A*
N2—H2···O2^i^	0.890 (10)	1.966 (12)	2.835 (3)	165 (2)

Symmetry codes: (i) *x*, −*y*+1/2, *z*−1/2.

## References

[bb1] Bruker (1998). *SMART*, *SAINT* and *SADABS* Bruker AXS Inc., Madison, Wisconsin, USA.

[bb2] Ejsmont, K., Zareef, M., Arfan, M., Bashir, S. A. & Zaleski, J. (2008). *Acta Cryst.* E**64**, o1128. 10.1107/S1600536808014645 PMC2961502 21202639

[bb3] Fun, H.-K., Patil, P. S., Jebas, S. R., Sujith, K. V. & Kalluraya, B. (2008). *Acta Cryst.* E**64**, o1594–o1595. 10.1107/S1600536808022861 PMC2962208 21203289

[bb4] Fun, H.-K., Patil, P. S., Rao, J. N., Kalluraya, B. & Chantrapromma, S. (2008). *Acta Cryst.* E**64**, o1707. 10.1107/S160053680802446X PMC2960631 21201695

[bb5] Jimenez-Pulido, S. B., Linares-Ordonez, F. M., Martinez-Martos, J. M., Moreno-Carretero, M. N., Quiros-Olozabal, M. & Ramirez-Exposito, M. J. (2008). *J. Inorg. Biochem.* **102**, 1677–1683. 10.1016/j.jinorgbio.2008.04.004 18538411

[bb6] Raj, K. K. V., Narayana, B., Ashalatha, B. V., Kumari, N. S. & Sarojini, B. K. (2007). *Eur. J. Med. Chem.* **42**, 425–429. 10.1016/j.ejmech.2006.09.010 17074422

[bb7] Sheldrick, G. M. (2008). *Acta Cryst.* A**64**, 112–122. 10.1107/S0108767307043930 18156677

[bb8] Yang, T., Cao, G.-B., Xiang, J.-M. & Zhang, L.-H. (2008). *Acta Cryst.* E**64**, o1186. 10.1107/S1600536808015912 PMC2961897 21202828

[bb9] Yehye, W. A., Rahman, N. A., Ariffin, A. & Ng, S. W. (2008). *Acta Cryst.* E**64**, o1824. 10.1107/S1600536808026846 PMC2960481 21201799

[bb10] Zhong, X., Wei, H.-L., Liu, W.-S., Wang, D.-Q. & Wang, X. (2007). *Bioorg. Med. Chem. Lett.* **17**, 3774–3777. 10.1016/j.bmcl.2007.04.006 17466518

